# Thiamine transporter-2 deficiency: outcome and treatment monitoring

**DOI:** 10.1186/1750-1172-9-92

**Published:** 2014-06-23

**Authors:** Juan Darío Ortigoza-Escobar, Mercedes Serrano, Marta Molero, Alfonso Oyarzabal, Mónica Rebollo, Jordi Muchart, Rafael Artuch, Pilar Rodríguez-Pombo, Belén Pérez-Dueñas

**Affiliations:** 1 Department of Child Neurology, Sant Joan de Déu Hospital, University of Barcelona, Passeig Sant Joan de Déu, 2, Esplugues, Barcelona 08950, Spain; 2 Department of Clinical Biochemistry, Sant Joan de Déu Hospital, University of Barcelona, Barcelona, Spain; 3 Department of Neuroradiology, Sant Joan de Déu Hospital, University of Barcelona, Barcelona, Spain; 4 Departamento de Biología Molecular, Centro de Diagnóstico de Enfermedades Moleculares (CEDEM), Centro de Biología Molecular Severo Ochoa CSIC-UAM, IDIPAZ, Universidad Autónoma de Madrid, Madrid, Spain; 5 Center for the Biomedical Research on Rare Diseases (CIBERER), ISCIII, Barcelona, Spain

**Keywords:** Thiamine transporter 2 deficiency, Biotin responsive basal ganglia disease, SLC19A3, Leigh syndrome, Lactic acidosis, Thiamine, Biotin, Striatal necrosis, Dystonia

## Abstract

**Background:**

The clinical characteristics distinguishing treatable thiamine transporter-2 deficiency (ThTR2) due to *SLC19A3* genetic defects from the other devastating causes of Leigh syndrome are sparse.

**Methods:**

We report the clinical follow-up after thiamine and biotin supplementation in four children with ThTR2 deficiency presenting with Leigh and biotin-thiamine-responsive basal ganglia disease phenotypes. We established whole-blood thiamine reference values in 106 non-neurological affected children and monitored thiamine levels in *SLC19A3* patients after the initiation of treatment. We compared our results with those of 69 patients with ThTR2 deficiency after a review of the literature.

**Results:**

At diagnosis, the patients were aged 1 month to 17 years, and all of them showed signs of acute encephalopathy, generalized dystonia, and brain lesions affecting the dorsal striatum and medial thalami. One patient died of septicemia, while the remaining patients evidenced clinical and radiological improvements shortly after the initiation of thiamine. Upon follow-up, the patients received a combination of thiamine (10–40 mg/kg/day) and biotin (1–2 mg/kg/day) and remained stable with residual dystonia and speech difficulties. After establishing reference values for the different age groups, whole-blood thiamine quantification was a useful method for treatment monitoring.

**Conclusions:**

ThTR2 deficiency is a reversible cause of acute dystonia and Leigh encephalopathy in the pediatric years. Brain lesions affecting the dorsal striatum and medial thalami may be useful in the differential diagnosis of other causes of Leigh syndrome. Further studies are needed to validate the therapeutic doses of thiamine and how to monitor them in these patients.

## Abstract (es)

**Resumen:**

**Antecedentes**

Las características clínicas distintivas del déficit tratable del trasportador de tiamina tipo 2 (ThTR2) debido a defectos genéticos del *SLC19A3* de las otras causas devastadores del síndrome de Leigh son escasas.

**Métodos**

Presentamos el seguimiento clínico después de la administración de suplementos de tiamina y biotina a cuatro niños con deficiencia ThTR2 que presentaban fenotipos de *biotin-thiamine responsive basal ganglia disease* y síndrome de Leigh. Hemos establecido valores de referencia de tiamina en sangre total en 106 niños sin patología neurológica y monitorizamos los niveles de tiamina en pacientes con mutación del *SLC19A3* después del inicio del tratamiento. Hemos comparado nuestros resultados con los de 69 pacientes con deficiencia ThTR2 después de una revisión de la literatura.

Al momento del diagnóstico , los pacientes tenían entre 1 mes a 17 años, y todos ellos mostraron signos de encefalopatía aguda, distonía generalizada, y lesiones cerebrales que afectan el cuerpo estriado dorsal y el tálamo medial. Un paciente murió de septicemia, mientras que el resto de pacientes evidenciaron mejoras clínicas y radiológicas poco después del inicio de la tiamina. Al seguimiento, los pacientes recibieron una combinación de tiamina (10–40 mg/kg/día) y biotina (1–2 mg/kg/día) y se mantuvieron estables, aunque con distonía y dificultades del habla residual. Después de establecer valores de referencia para los diferentes grupos de edad, la cuantificación de tiamina en sangre total demuestra ser un método útil para el seguimiento del tratamiento.

**Conclusiones**

La deficiencia ThTR2 es una causa reversible de la distonía aguda y síndrome de Leigh en la edad pediátrica. Las lesiones cerebrales que afectan el cuerpo estriado dorsal y tálamo medial pueden ser útiles en el diagnóstico diferencial de otras causas de síndrome de Leigh. Se necesitan más estudios para validar las dosis de tiamina y la monitorización terapéutica de estos pacientes.

## Background

Acute encephalopathy with bilateral striatal necrosis in childhood includes several disorders of infectious, autoimmune, metabolic and genetic origin [1-5]. One of these diseases is thiamine transporter-2 deficiency (ThTR2, OMIM**#**607483), a recessive inherited defect due to mutations in the *SLC19A3* gene that cause acute and recurrent episodes of encephalopathy with dystonia, seizures and brain injury that respond extremely well to the early administration of thiamine and biotin [6-20]. However, biochemical or neuroimaging criteria for diagnosis are not available, and timely and effective treatment relies on a high index of clinical suspicion. Of particular interest is the distinction of ThTR2 from other untreatable causes of Leigh syndrome, such as defects in the nuclear and mitochondrial genes encoding components of the oxidative-phosphorylation system or the pyruvate metabolism, causing a devastating disorder with similar clinical and radiological features in the pediatric age.

We aim to describe the phenotypes of four children with mutations in the *SLC19A3* gene, comparing their clinical, biochemical, radiological and genetic data with all of the formerly reported patients and discussing the possible clinical and radiological clues for the distinction of ThTR2 from other causes of irreversible basal ganglia necrosis, especially Leigh syndrome. Moreover, we report the follow-up after thiamine and biotin supplementation and the utility of monitoring whole-blood thiamine concentrations.

## Methods

### Patients

Four children with *SLC19A3* gene mutations were diagnosed at a tertiary university children’s hospital (Hospital Sant Joan de Déu, University of Barcelona) during the past 4 years.

Patients 1, 3 and 4 were diagnosed at the onset of acute encephalopathy and received early treatment with thiamine and biotin. A clinical description of these patients at diagnosis has been previously reported [11,19]. Patient 2 was identified by mutation screening for the *SLC19A3* gene in 11 children with Leigh syndrome who had normal respiratory chain enzyme analyses. Leigh patients were previously analyzed for mitochondrial DNA mutations and for candidate nuclear genes associated with Leigh syndrome, all with negative results. The patients were evaluated using a standardized protocol, including a complete physical and neurological examination and biochemical studies at diagnosis, at 4 weeks and every 6 months after the onset of encephalopathy. Brain MRIs were performed at diagnosis and at 6 to 12 months after the initiation of treatment. Samples were obtained in accordance with the Helsinki Declaration of 1964, as revised in October 2013 in Fortaleza, Brazil. Ethical permission for the studies was obtained from the Research & Ethics Committee of the Hospital Sant Joan de Déu.

### Neuroimaging

MRI examinations were performed on a 1.5-T magnet system (Signa Excite HD, Milwaukee, WI, USA), obtaining a sagittal T1-weighted, axial fast-spin echo with fluid-attenuated inversion recovery (FLAIR) and T2-weighted imaging. Diffusion weighted imaging was performed in patient 1. Two pediatric neuro-radiologists reviewed the images.

### Laboratory studies

Blood concentrations of lactate and pyruvate were measured by standard automated spectrometric procedures. Plasma amino acids and urinary organic acids were analyzed following previously reported procedures [21,22].

The concentration of thiamine and its metabolites (thiamine monophosphate (TMP) and thiamine diphosphate (TDP)) were analyzed in whole-blood EDTA samples by high-performance liquid chromatography (HPLC) with fluorescence detection (Perkin Elmer, series 200, Norwalk, CT, USA) according to a modified reported procedure [23]. Whole-blood thiamine, TMP and TDP reference values were established in 106 children (59% males) referred to our hospital for minor surgical interventions. Exclusion criteria were the presence of chronic diseases, malnutrition and special diets. Whole-blood thiamine was quantified in patients 1, 2 and 3 at 6 months and 12 months after the onset of treatment.

### Molecular analysis of the *SLC19A3* gene

Genomic DNA from the blood samples of 11 patients with Leigh syndrome were used for the mutation analysis of the *SLC19A3* gene (RefSeq accession number NM_025243.3_ [mRNA]). The coding region and the flanking intron-exon boundaries were PCR amplified with primers based on the Ensembl genome browser entry ENSG00000135917. The amplicons were sequenced and analyzed as previously described [24]. The mutation nomenclature used follows that described at http://www.hgvs.org./mutnomen/.

### Systematic review of the literature

We searched MEDLINE (through PubMed) using the following keywords: #1 SLC19A3, #2 thiamine transporter-2, #3 Leigh encephalopathy, #4 ThTR2 and #5 biotin responsive basal ganglia disease. The number of hits at 02/01/2014 was 50, 190, 44, 5, and 14, respectively. A total of 15 clinical studies (4 case reports, 11 quantitative series) and 1 guideline/clinical practice proposal were finally selected [6-20].

## Results

### Patients

Table 1 summarizes the clinical, biochemical and genetic data of the four patients with *SLC19A3* defects.

**Table 1:** The clinical, biochemical and genetic data of the four patients with thiamine transporter-2 deficiency

**Patients**	**1**	**2**	**3**	**4**
**Origin**	**Morocco**	**Spain**	**Spain**	**Spain**
Mutation *SLC19A3* gene	c.68G < T in homozygosis	c.1079dupT/ c.980-14A < G	c.74dupT/ c.980-14A < G	c.74dupT/ c.980-14A < G
Phenotype	Leigh syndrome	Leigh syndrome	BTBGD	BTBGD
Sex and Onset	Male, 1 month	Male, 13 months	Female, 4 years	Male, 15 years
Encephalopathy	Lethargy, vomiting	Irritability, continuous crying	Agitation, lethargy	Agitation, coma
Extrapiramidal features	Hypotonia, jitteriness, dystonia, opisthotonus, tremor	Hypotonia, status dystonicus, ophistotonus, tremor	Paroxysmal dystonia, generalized dystonia, tremor	Status dystonicus, akinetic-rigid syndrome, tremor
Cranial nerves	Dysphagia	Dysphagia, nystagmus, strabismus	Anarthria, dysphagia	Nystagmus, ptosis, diplopia, dysarthria, vertigo, facial dyskinesias/hyposthesias
Others	Pyramidal signs	Ataxia, weight loss, hepatomegaly, jaundice	None	Pyramidal signs, rabdomyolisis, dysautonomia, generalized seizures
Plasma Lactate (RR 0,7 – 2,4 mmol/L)	8.6	2.3	1.6	1.2
Plasma Pyruvate (RR 0,03-0,1 mmol/L)	0.14	0.1	0.21	0.13
Lactate/Pyruvate ratio (RR 11–30)	19.1	23.4	11.5	18.2
Alpha Alanine (RR 167 – 439 μmol/L)	637	355	300	370
CSF Lactate (RR 1,1 – 2,2 mmol/L)	7.1	1.7	Not performed	1.8
Organic acid analysis in urine	High excretion of alpha-ketoglutarate (11463 mmol/mol creatinine)	High excretion of 2-hydroxy acids, isobutyric, 2-hydroxy-isovaleric acid, 2,4-dihydroxybutyric	Normal	Normal

BTBGD: Biotin-thiamine responsive basal ganglia disease.

Four patients suffering *SLC19A3* mutations had no relevant family history for neurological diseases and were normally developing children until the onset of symptoms (mean age 3 years, range 1 month - 8 years). A trigger condition before the neurological episodes was identified in patients 2 and 4 (gastroenteritis, trauma, strong physical exercise and an upper respiratory tract infection).

All of the patients showed signs of encephalopathy and focal or generalized dystonia. In all of the cases, dystonia progressed to be generalized, and 2 patients had associated *opisthotonus*. Patients 2 and 4 suffered *status dystonicus* and were transferred to the intensive care unit for profound sedo-analgesia. Patient 2 received diazepam, levomepromazine and chlorpromazine, followed by midazolam from day 27 to day 34, when he died. Patient 4 received trihexyphenidyl and diazepam for several days until the spasm and posture were under control.

Other clinical features at onset of the disease are reported in Table 1.

Clinical improvement was evidenced shortly after the initiation of thiamine in patients 1, 3 and 4 (the daily doses varied from 15 to 30 mg/kg/day and were given orally in two or three divided doses), combined with biotin in patient 1 (10 mg/day). Patient 2 suffered septicemia caused by *Enterobacter cloacae* and hepatic and cardiac failure and died 34 days after admission. Thiamine was empirically initiated 6 days before he died (initial doses of 150 mg daily, followed later by 1 g, twice a day).

### Patient follow-up

Currently, patients 1, 3 and 4 are 25 months, 8 years and 23 years old, respectively. The median follow-up of these patients is 57 months (range 22 – 99 months). As of the last visit, they are receiving a combination of thiamine (10 – 40 mg/kg/d) and biotin (1 – 2 mg/kg/d) (Table 1), and they remain stable under this treatment and have not suffered any new episodes of encephalopathy.

Patient 1 developed independent gait at 19 months, and on his last examination at the age of 25 months he was walking, with occasional falls due to gait-induced dystonia. He plays and eats independently, but upper limb dystonia and thumb adduction partially interfere with his fine motor skills. He has oro-mandibular dystonia and expressive language delay, but his language comprehension and cognitive skills are in the average range for his age. A physical examination also showed pyramidal signs in the lower limbs. He has begun an intensive physiotherapy program.

Patient 3 is asymptomatic at 8 years old, and her neurological examination is normal. She attends a normal school and achieves good academic performance. She developed a nephrotic syndrome at 6 years old that was responsive to oral corticosteroids.

Patient 4 is 23 years old and has mild dysarthria and dysphagia. He shows intermittent facial dyskinesia and eye-blinking, as well as dystonic posturing of his right upper limb and left foot. He has some difficulties with activities that require fine motor skills, such as buttoning, tying shoes or opening bottles. He is engaged in gainful employment and exercises regularly.

### Neuroimaging

**Figure 1:**
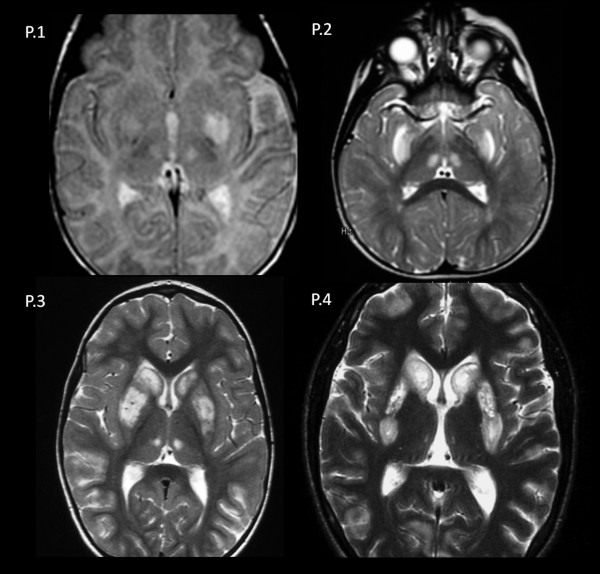
**Axial T2 FSE demonstrated bilateral and symmetrical involvement of the putamina and medial thalamic nuclei in patients 1 (P.1), 2 (P.2), 3 (P.3) and 4 (P.4).** In patients 2, 3 and 4, the head of the caudates were also affected.

**Figure 2:**
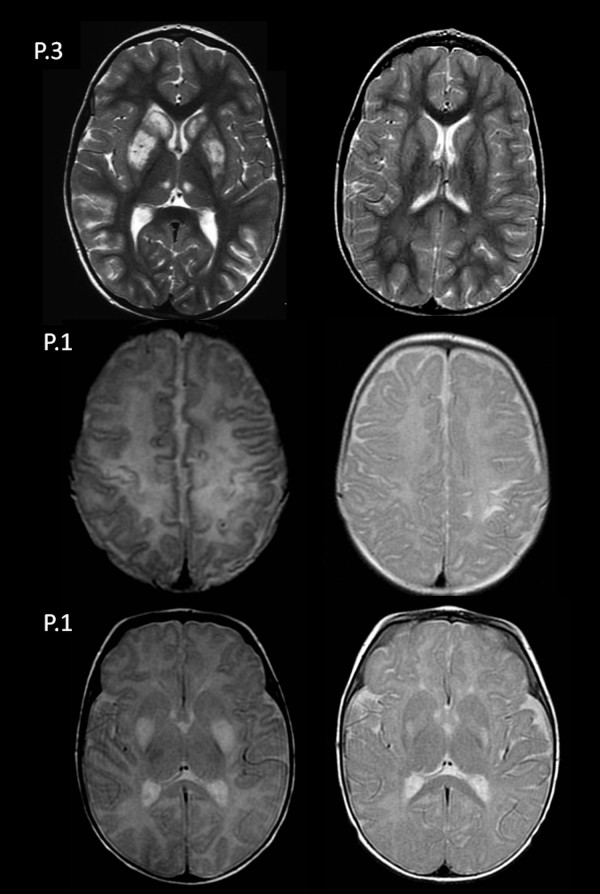
**Axial T2 FSE of patient 3 (P.3) at the level of the basal ganglia and of patient 1 at the level of the peri-rolandic region (P.1) and the basal ganglia (P.1) before and after treatment.** There is a dramatic improvement of the lesions after thiamine supplementation.

The brain MRIs of the four patients in the acute phase showed lesions in both the dorsal striatum and the medial thalamic nuclei (Figure 1). Putamen involvement was diffuse in patients 2, 3 and 4 and was limited to the posterior region in patient 1. A concentric lesion of the head of the caudate was observed in patients 2, 3 and 4. There was variable cortical and subcortical involvement of the hemispheres: in patient 1, lesions had a peri-rolandic distribution; in patients 2 and 4, they were patchily distributed across both cerebral hemispheres. Diffusion weighted imaging was performed in patient 1 showing low ADC values in the putamina and peri-rolandic cortex. High lactate peaks were detected in patients 2 and 4 on MR spectroscopy.The follow-up MRIs performed at age 6 months (patient 1), 7 years (patient 3) and 20 years (patient 4) showed an improvement in the signal abnormalities in all of the patients (Figure 2). Residual abnormal signal intensity and volume loss were observed in the putamen (patients 1, 3 and 4) and head of the caudate (patients 3 and 4). The cortical and subcortical lesions disappeared in patient 3, but volume loss was observed at the peri-rolandic region in patient 1 (Figure 2).

### Laboratory studies

**Figure 3:**
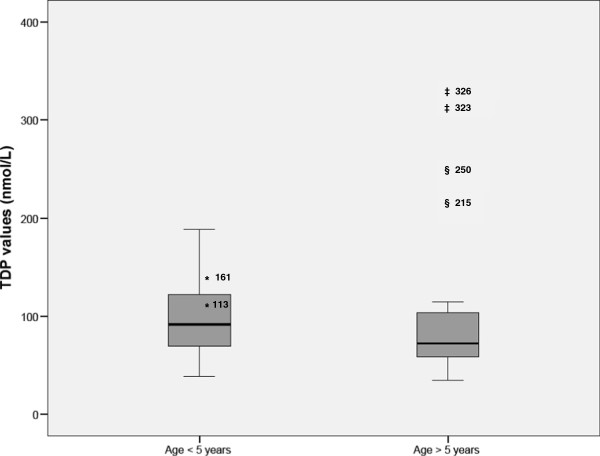
**Box-plot representations of the whole-blood TDP concentrations divided into two intervals: > 5 years (n = 67): 90.3 nmol/L (38.8-188.4) (median, range); < 5 years (n = 39): 68.8 (34.2-114.8) (median, range).** The Mann–Whitney U test showed significantly different values for TDP when comparing both groups (U = 731, p > 0.001). The TDP values of patients 1(*), 2 (‡) and 3 (§) under thiamine treatment are also represented in the figure. The length of the boxes indicates the interquartile space (P25-P75), the horizontal line represents the median (P50), and the bars indicate the range.

**Figure 4:**
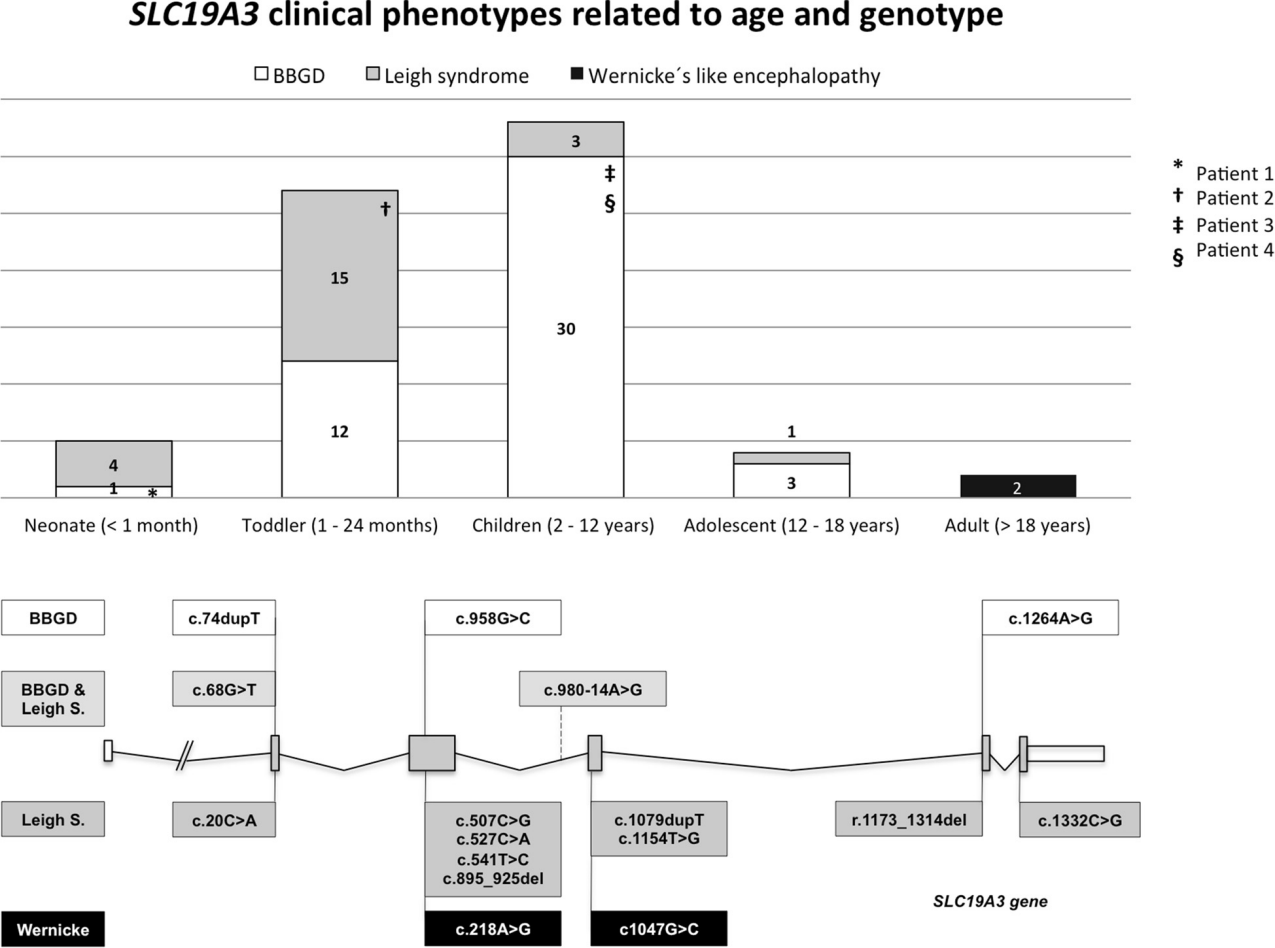
**The upper figure shows a distribution of the age at onset in all reported patients with ThTR2 deficiency and the related clinical phenotypes.** The lower figure shows a schematic representation of all reported *SLC19A3* mutations and the related clinical phenotypes.

The biochemical analysis at diagnosis showed high lactate levels in patient 1 (Table 1). Patient 2 had normal lactate concentrations until he presented with septicemia, when lactic acid increased to 16 mmol/L. Alanine was increased only in patient 1. Organic acids showed high excretion of alpha-ketoglutarate in patient 1 and mild excretion of 2-hydroxy acids, isobutyric, 2-hydroxy-isovaleric acid and 2,4-dihydroxybutyric in patient 2.The analysis of thiamine, TDP and TMP isoforms in whole-blood samples in the control patients showed that the TDP isoform represented 85% of the whole thiamine concentration. Therefore, TDP values were used for treatment monitoring. The reference whole-blood TDP values were stratified into two age groups, as a statistically significant negative correlation was observed between whole-blood TDP values and the age (r = −0.290; p = 0.003) (Figure 3).On the last follow-up visit, Patient 3 was taking biotin (2 mg/kg/day) and thiamine (10 mg/kg/day) and patient 4 was taking biotin (2 mg/kg/day) and thiamine (15 mg/kg/day). Both patients had TDP values above the upper limit of our reference range (Figure 4). Patient 1 was receiving biotin (1.2 mg/kg/day) and thiamine (40 mg/kg/day), but his TDP concentrations did not reach the upper limit of the age reference range. Persistent lactic acidemia (mean 2.69 mmol/L, range 2.1–3.46) was detected in the follow-up of this patient.

### Molecular studies

A Sanger sequencing of the *SLC19A3* gene in patients 1, 3 and 4 had identified missense, small duplication and splicing mutations, all of which were carried either in a homozygous or heterozygous fashion [11,19]. The mutation analysis in Patient 2 disclosed two different changes, both of which created premature stop codons in the ThTR2 protein sequence. One of the changes was the previously described c. 980-14A < G, and the other change was the novel duplication, c.1079dupT, with a predictable effect on the protein of p.Leu360Phefs*38. A schematic representation of the *SLC19A3* mutations present in our four patients and in all previously reported patients is shown in Figure 4.

### Review of the literature

A summary of the clinical data from the literature review in 69 patients is reported in Table 2. Most of the patients (80%) presented with symptoms before the age of 12 years (age onset 3.5 ± 4.6 years (mean ± SD), range 1 month – 20 years). The patients exhibited the following phenotypes: biotin-thiamine responsive basal ganglia disease (BTBGD) (N = 46), Leigh encephalopathy (N = 23) and Wernicke encephalopathy (N = 2). Figure 4 shows the age at onset of all of the formerly reported patients, as well as their clinical phenotypes and related genotypes.

**Table 2:** The clinical and radiological features of patients with thiamine transporter-2 deficiency reported in the literature

	**Number of patients**		**Number of patients**
Patients	69	Dead patients	23
Age (years ± SD)	3.5 ± 4.3	**Symptoms at follow up**	
Male/Female	36/33	Tetraparesia/Dystonia	32
Trigger events	40	Cognitive impairment	23
**Symptoms at onset**		Dysphagia	13
Encephalopathy/Lethargy	57	Epilepsy	11
Seizure	47	Dysarthria	10
Generalized and focal dystonia	38	Respiratory support	4
Dysarthria/Anarthria	28	Ataxia	3
Ataxia	25		
Dysphagia	21	**MRI**	
Pyramidal signs	19	Caudate	55
Abnormal ocular movement	17	Putamen	55
Developmental delay	12	Thalami	31
Opisthotonus	11	Cerebellum	22
Rigidity/Rigid akinetic syndrome	11	Brainstem	19
Tremor	4	Subcortical WM	16
Chorea	2	Cerebral cortex	13
Jitteriness	2	Globus pallidus	8
Dystonic status	2	Medulla	3
Dysautonomia	2	Lactate on spectroscopy	6
Ptosis	2		

The table shows a list of signs and symptoms at onset and at follow-up, as well as MRI abnormalities. Seizures include myoclonic jerks, epileptic spams, focal and generalized seizure, *epilesia partialis continua* and *status epilepticus*. Abnormal ocular movements include nystagmus, oculogyric crisis, oculomotor nerve palsy, ophtalmoplegia and sunset phenomenon. Symptoms reported only once: rhabdomyolisis, facial dyskinesia. SD: Standard Deviation.

In regard to treatment, there were some reports of using biotin alone (N = 2) [6,9,10], thiamine alone (N = 3) [8,13], and biotin combined with thiamine (N = 5) [10,11,14,16,18,19]. In two studies on Leigh patients, the diagnosis was performed retrospectively, and treatment with vitamins was introduced late in the evolution of symptoms in a few patients, with very poor outcomes [12,13].

## Discussion

We describe four patients with ThTR2 deficiency presenting with acute encephalopathic episodes and generalized dystonia between 1 month and 15 years of age. Their dystonia was improved when each of them were administered thiamine, with the exception of the patient treated late in the evolution of the disease. The data from the previously reported patients with *SLC19A3* mutations showed that either focal or generalized dystonia, in combination with decreased consciousness and seizures, were the most common clinical features at onset and were reported in more than fifty percent of the patients [6-20], reflecting that ThTR2 deficiency is an important cause of reversible dystonia in children. Hence, a trial with thiamine should be indicated in every case of acute dystonia. Patients also presented with other less common extrapyramidal and pyramidal features, cranial nerve palsy, dysautonomia, rhabdomyolisis, jaundice and other systemic symptoms.

The literature review showed that most patients with *SLC19A3* mutations experienced an onset of the disease between 1 month and 12 years of age. Two-thirds of the patients were classified as BTBGD and the remaining patients were classified as having Leigh and Wernicke encephalopathies. However, there is probably a clinical continuum among patients that, in view of the reported mutational spectrum, appears to be biologically more plausible. In fact, patient 2 with Leigh syndrome in our series carried the mutation c.980-14A < G, which has been previously described in children with a BTBGD phenotype [10,11], and patient 1 who also presented with infantile lactic acidosis and Leigh syndrome harbored the mutation c.68G < T, which has been previously associated with BTBGD [7]. We also observed that patients with the same mutations had different ages at onset (e.g., c.1264A < G [14,15] and c.20C < A [13]). Considering the dense genetic interaction network that sustains a disease phenotype, it is probable that a combination of yet unknown genetic and environmental factors may be responsible for the different age presentations and related phenotypes [25,26].

Despite the genetic heterogeneity of our patients and the wide age range of disease at onset, all of them presented symmetrical involvement of the dorsal striatum and medial thalamic nuclei. In the older patients, the head of the caudate was always affected, and the cortical and subcortical lesions showed a diffuse and patchy distribution in the cerebral hemispheres [14,15]. In the newborn, the caudate was not affected, and there was a selective involvement of the peri-rolandic area [19]. The differences in the distribution of the brain lesions observed in our patients probably depend on the regional variations in the energetic demands according to the different ages. Although this pattern of brain lesions may not be specific, it can be useful in suggesting the diagnosis of a *SLC19A3* defect. In line with our results, the literature review showed that the most frequent brain areas involved in ThTR2 deficient patients were, in order of frequency, the caudate, putamen and thalamus, followed by the cerebellum, brainstem and cerebral hemispheres [6-20].

Regarding the biochemical findings, lactic acidemia and high excretion of organic acids were detected during acute metabolic decompensations in two infants in our series. Thiamine is an essential cofactor of 3 mitochondrial enzymes: pyruvate dehydrogenase complex, alpha-ketoglutarate dehydrogenase, and branched-chain alpha-keto acid dehydrogenase. These enzymes are involved in the oxidative decarboxylation of pyruvate, alpha-ketoglutarate, and branched chain amino acids, respectively. The biochemical abnormalities detected in our patients could be due to the decreased activity of these thiamine-dependent mitochondrial enzymes [19]. The older children showed normal biochemical analyses for plasma, urine and CSF, and lactic acid accumulation was detected only on MR spectroscopy. Similarly, other authors have described high amounts of lactic acid in the serum and high excretion of organic acids in the urine of patients with fatal infantile Leigh phenotypes (Table 1) [12,13] but normal biochemical profiles in children classified as having BTBGD phenotypes [8-10,14].

We observed a dramatic response to high doses of thiamine in the three patients who were treated during the first days of encephalopathy. Clinical follow-up showed a complete clinical and radiological recovery in one patient, but the other two patients showed residual dystonia, speech difficulties, and necrotic changes in the dorsal striatum and the frontal cortex.

Even though thiamine was initiated four weeks after the onset of symptoms, patient 2 died at 14 months of age. In other reported cases of fatal infantile Leigh and *SLC19A3* defects, the lesions progressed to cystic degeneration and severe atrophy, suggesting that the prognosis of these patients is poor and largely depends on the early administration of biotin and thiamine [8,10,15]. Although *SLC19A3* deficiency is considered to be a treatable entity, the literature review showed that sixty percent of previously reported patients with either BTBGD or a Leigh phenotype had poor outcomes, including early death, tetraparesis, dystonia or cognitive impairment. At the other end of the spectrum, patients with Wernicke encephalopathy showed lesions that selectively affected the periaqueductal grey matter, which disappeared when thiamine was initiated [8].

When establishing the whole-blood thiamine reference values, we found that TDP was the most concentrated thiamine isoform, similar to other studies [23,27]. For this reason, treatment monitoring relied on whole-blood TDP concentrations. Patients treated with 10 to 40 mg/kg/day of thiamine were clinically stable for a mean follow-up of 57 months. At these doses, TDP levels remained above the upper limit of the reference values in patients 3 and 4. Conversely, in patient 1, the TDP concentrations remained in the reference range, and he presented persistent acidosis. These data led to the suspicion of poor family adherence to the treatment, which was confirmed and corrected with the participation of a social worker in the follow-up program. This patient did not present any clinical relapse, even though lactic acid concentrations were persistently elevated, perhaps due to the absence of relevant trigger factors during follow-up.

Currently, there is no agreement in the long-term doses of vitamins that should be administered in *SLC19A3* deficient patients, and the documented doses of thiamine and biotin vary from 100 to 900 mg per day and from 2 to 12 mg/kg per day, respectively [6-20]. It is likely that higher doses are required when trigger factors, such as fever or trauma, are present [18]. Initial reports described a good response to biotin as a monotherapy [8]. However, a recent description by Tabarki et al. reported that a high proportion of patients treated with biotin only showed recurrences of encephalopathy compared with those who received biotin and thiamine simultaneously [14].

We detected pathogenic mutations in the *SLC19A3* gene in 1 of 11 patients with Leigh syndrome. Similarly, Gerards et al. reported that 2 of 17 Leigh patients were positive for *SLC19A3* mutations [13]. The MRI pattern of brain injury involving the dorsal striatum and medial thalamic nuclei in patients with SLC19A3 defect may be useful to distinguish this disorder from other causes of Leigh syndrome. Interestingly, some correlations have been described between MRI findings and specific genetic defects. In patients with *ATPase 6* mutations MRI typically shows necrosis in the putamina, demyelization in the corona radiata and cerebellar and brainstem atrophy in the final stages [28]. Patients with PDHc deficiency usually present with lesions in the basal ganglia, brainstem and dentate nuclei, being the globus pallidus frequently involved [29]. A common pattern of brain MRI in patients with Complex I deficiency consists of brainstem and striatal lesions (putamina more frequently than the caudate and pallidum) [30]. MRS may show lactate peaks during the acute phase in *SLC19A3* defects and in other causes of Leigh syndrome [11].

In view of the overlapping phenotypes that may exist between ThTR2 deficiency and mitochondrial disorders causing Leigh encephalopathy, it seems advisable to initiate empirically biotin and thiamin in every patient with Leigh syndrome. However, it is concerning that in a recent report on the practice patterns of mitochondrial disease physicians in North America, only 3 of 32 medical doctors administered thiamine and other B complex vitamins [31].

In conclusion, thiamine transporter-2 deficiency is an inherited recessive disease that affects the central nervous system during development and may present as Leigh syndrome in infants, mimicking untreatable mitochondrial disorders. A characteristic MRI pattern of caudate, putamen and medial thalamus involvement, in association with lactic acid accumulation and high excretion of organic acids in urine in infants, suggests the diagnosis. It is of utmost importance to start early treatment with thiamine and biotin because the process may be at least partially reversible. Currently, there is an urgent need for validated tools for early diagnosis and treatment monitoring. In our experience, thiamine quantification by the HPLC method in whole-blood samples appears to be a useful method for the evaluation of the adherence to treatment. Further studies are needed to validate the therapeutic doses of thiamine and how to monitor them in these patients.

## Abbreviations

DNA: Deoxyribonucleic acid; HPLC: High-performance liquid chromatography; MRI: Magnetic resonance image; MRS: Magnetic resonance spectroscopy; BTBGD: Biotin-thiamine responsive basal ganglia disease; FLAIR: Fluid attenuated inversion recovery; TMP: Thiamine monophosphate; TDP: Thiamine diphosphate; PCR: Polymerase chain reaction; ThTR2: Thiamine transporter type 2; CSF: Cerebrospinal fluid; HIE: Hypoxic ischemic encephalopathy.

## Competing interests

The authors declare that they have no competing interest.

## Authors’ contributions

JDOE conceptualized and designed the study, drafted the initial manuscript, and approved the final manuscript as submitted. MS contributed to the analysis and interpretation of the clinical data, critically reviewed the manuscript, and approved the final manuscript as submitted. MM contributed to the analysis and interpretation of the biochemical studies, critically reviewed the manuscript, and approved the final manuscript as submitted. AO contributed to the analysis and interpretation of the molecular studies, critically reviewed the manuscript, and approved the final manuscript as submitted. MR contributed to the analysis and interpretation of the neuroradiological studies, critically reviewed the manuscript, and approved the final manuscript as submitted. JM contributed to the analysis and interpretation of the neuroradiological studies, critically reviewed the manuscript, and approved the final manuscript as submitted. RA contributed to the analysis and interpretation of the biochemical studies, critically reviewed the manuscript, and approved the final manuscript as submitted. PRP contributed to the analysis and interpretation of the molecular studies, critically reviewed the manuscript, and approved the final manuscript as submitted. BPD conceptualized and designed the study, contributed to the analysis and interpretation of the results, critically reviewed the manuscript, and approved the final manuscript as submitted.
